# Perceived epilepsy-related support and enabling environments and associated caregiver factors in rural South Africa: a cross-sectional study in Limpopo and Mpumalanga

**DOI:** 10.3389/fpubh.2026.1881761

**Published:** 2026-09-07

**Authors:** Happiness Ngobeni, Thendo Gertie Makhado, Lufuno Makhado

**Affiliations:** 1 Department of Public Health, Faculty of Health Sciences, University of Venda, Thohoyandou, South Africa; 2 Department of Advanced Nursing Sciences, Faculty of Health Sciences, University of Venda, Thohoyandou, South Africa

**Keywords:** caregiving burden, epilepsy, family acceptability, family caregivers, people with epilepsy, rural health, social inclusion, South Africa

## Abstract

**Background:**

Family caregivers are important sources of support for people with epilepsy (PWE), particularly in rural settings where formal services may be limited. However, less is known about caregivers' perceptions of the community and the informational and service conditions that support the inclusion of PWE.

**Objective:**

This study described caregivers' perceptions of epilepsy-related support and enabling environments and examined the caregiver and contextual factors associated with these perceptions in selected rural communities in Limpopo and Mpumalanga, South Africa.

**Methods:**

A cross-sectional study was conducted among 384 family caregivers of PWE in Collins Chabane Local Municipality, Limpopo, and Bushbuckridge Local Municipality, Mpumalanga. Data were collected using a self-developed questionnaire covering stigma, caregiving burden, barriers, and support or enabling conditions. The instrument underwent an exploratory ordinal psychometric assessment. Perceived epilepsy-related support and enabling conditions were measured using a four-item continuous score ranging from 4 to 20. The score demonstrated acceptable preliminary ordinal reliability (ordinal α = 0.723; McDonald's ω = 0.729). Multivariable linear regression with heteroscedasticity-robust standard errors was used to identify associated factors.

**Results:**

The mean age of respondents was 49.4 years (SD = 14.6; median = 49; IQR = 39–60; range = 18–86). The mean support and enabling-environment score was 6.52 (SD = 2.77), indicating limited availability of the measured support structures. Caregivers at the Mpumalanga study site had scores that were, on average, 1.13 points lower than those at the Limpopo site after adjustment (B = −1.13; 95% CI −1.71 to −0.54; *p* < 0.001). A greater emotional and safety-related caregiving burden was associated with a lower support-environment score (B = −0.31; 95% CI = −0.44 to −0.18; *p* < 0.001). Age was not independently associated with the continuous outcome (B = −0.010; 95% CI = −0.032 to 0.012; *p* = 0.377). The positive association between barriers and the support-environment score was not interpreted substantively because shared item wording and response style may have contributed to this relationship.

**Conclusion:**

Caregivers reported limited access to support groups, epilepsy management information, community leadership support and organized community inclusion mechanisms. The findings describe perceptions of the contextual support environment rather than direct family acceptance of PWE. Further development and independent validation of the questionnaire are required.

## 1 Introduction

Epilepsy is a chronic neurological disorder characterized by recurrent unprovoked seizures resulting from abnormal electrical activity in the brain. It affects people of all ages and has clinical, psychological and social consequences that extend beyond seizure occurrence. Approximately 80% of people with epilepsy live in low- and middle-income countries, where access to diagnosis, antiseizure medication and continuing care remains uneven. Although appropriate treatment could enable up to 70% of PWE to become seizure-free, about three-quarters of those living in low-income countries do not receive the treatment they require (1).

The effects of epilepsy extend to families and communities. PWE may experience discrimination, social exclusion and restrictions in education, employment, relationships and community participation. Their families may also experience stigma, social isolation and economic strain. Misconceptions that epilepsy is contagious, caused by witchcraft or linked to supernatural forces, remain evident in parts of sub-Saharan Africa and can contribute to fear, concealment, social distancing and delayed use of biomedical care (2). Recognition of epilepsy as a medical condition may coexist with cultural and spiritual explanations, demonstrating that biomedical knowledge alone does not necessarily eliminate stigmatizing beliefs. The International League Against Epilepsy has consequently emphasized that epilepsy-related stigma is multidimensional and requires interventions at individual, family, community and structural levels (3).

Family members frequently provide practical, emotional, supervisory and financial assistance to PWE. Their responsibilities may include supporting medication adherence, accompanying the person to health services, responding to seizures and reducing the risk of injury. However, not every PWE requires continuous caregiving, and the nature and intensity of assistance may vary with seizure frequency, functional ability, treatment response, household circumstances and available resources. For relatives who assume a key caregiving role, the unpredictability of seizures can contribute to persistent vigilance, fear, uncertainty and emotional exhaustion. Evidence indicates that epilepsy caregivers may experience psychological distress, disruption of social and occupational activities and unmet needs for epilepsy-related information and psychosocial support (4, 5). Poor social support and epilepsy-related stigma have also been associated with greater psychological distress among caregivers (6).

A supportive environment may strengthen caregivers' ability to respond to these challenges. Such an environment may include accessible health services, reliable epilepsy-management information, caregiver support groups, inclusive community leadership and community participation in stigma reduction. The World Health Organization's *Intersectoral Global Action Plan on Epilepsy and Other Neurological Disorders 2022–2031* calls for person-centered care, caregiver support, reduced stigma and collaboration between healthcare services, civil society and community stakeholders (7). These contextual conditions are distinct from direct family acceptance. Family acceptance concerns how relatives regard, include and behave toward a PWE, whereas an enabling environment concerns the external resources and social conditions that support PWE and their caregivers.

The study was informed by a multilevel understanding of epilepsy-related support, recognizing that family caregiving occurs within interconnected household, community, cultural, informational and health-service environments. Caregiver experiences may be shaped by caregiving demands, access to health services and reliable information, community beliefs, social networks and the involvement of community leadership (2–4, 7). These influences may operate simultaneously: caregivers can experience emotional or practical burden while also encountering stigma, service barriers or varying levels of community support. Accordingly, stigma-related experiences, caregiving burden, health-system and community barriers, and contextual support were assessed as distinct but potentially interrelated domains rather than being combined into a single acceptability construct.

Research in rural Limpopo and Mpumalanga has identified gaps in epilepsy knowledge, attitudes and seizure-management practices among caregivers and family members, highlighting the need for culturally appropriate education and community-based interventions (8). However, less is known about caregivers' perceptions of the practical support and enabling conditions available within these rural settings or the factors associated with those perceptions. This study therefore assessed caregivers' perceptions of epilepsy-related support and enabling environments in selected rural communities in the Limpopo and Mpumalanga provinces of South Africa. It also examined whether these perceptions were associated with study location, caregivers' sociodemographic characteristics, practical, social and financial burden, emotional and safety-related burden and perceived health-system and community barriers.

## 2 Material and methods

### 2.1 Study design

This quantitative cross-sectional study was conducted in Bushbuckridge Local Municipality, Mpumalanga Province and Collins Chabane Local Municipality, Limpopo Province, South Africa. Both municipalities contain predominantly rural communities in which primary healthcare facilities are important sources of epilepsy care, and access to specialized neurological services is limited.

### 2.2 Study setting

The study was undertaken in purposively selected rural municipalities in Limpopo and Mpumalanga. These settings were selected because epilepsy had been identified as an important community health concern and because the study team had access to local structures that could support participant identification and recruitment.

The purposive selection of the sites means that the sample should not be regarded as representative of all caregivers of PWE in either province. The criteria and local arrangements used to identify high-burden communities may also have differed between the selected sites. Consequently, comparisons between provinces reflect differences across the selected study settings and should not be interpreted as population-level provincial effects.

The total number of PWE residing in the selected study sites was not determined because no complete epilepsy register or reliable population-based enumeration of PWE was available for these communities.

### 2.3 Study population

The study population consisted of adult key family caregivers of PWE residing in selected rural communities in the Limpopo and Mpumalanga provinces of South Africa. A key caregiver was defined as the adult household or family member who assumed the principal responsibility for providing practical, emotional, supervisory or health-related support to a PWE. Only one key caregiver was included per household and per PWE. Where more than one family member provided assistance, the household identified the person with the primary caregiving role. Multiple caregivers representing the same PWE were not enrolled. Eligible participants were adults involved in the day-to-day care or support of a person with epilepsy and who provided informed consent to participate. Individuals who were unwilling or unable to complete the questionnaire were excluded from the study. The study was conducted in Bushbuckridge Local Municipality, Mpumalanga Province and Collins Chabane Local Municipality, Limpopo Province. The combined population of the two municipalities was estimated at 912,191, comprising 564,216 residents in Bushbuckridge and 347,975 in Collins Chabane. These municipal population figures describe the broader study context and were not used as the denominator for calculating the caregiver sample. The number of PWE and eligible key caregivers residing in the two municipalities was not determined.

The study included caregivers of persons with any type of epilepsy. No eligibility restrictions were applied based on epilepsy type, seizure classification or severity. The clinical severity of epilepsy, seizure frequency, level of functional dependence and ability of the PWE to perform activities of daily living were not independently assessed. The study, therefore, represents the experiences of family members who identified themselves as key caregivers.

### 2.4 Eligibility criteria

Participants were included if they:

- were aged 18 years or older;
- were identified as the key family or household caregiver of a PWE;
- resided in one of the selected study sites;
- were able to provide informed consent; and
- were willing to participate.

Caregivers were excluded if another caregiver from the same household or representing the same PWE had already been enrolled.

### 2.5 Sample-size determination

The required sample size was calculated using the single-population proportion formula:


[n=\frac{Z ^2p(1−p)}d ^2}]

A 95% confidence level, an assumed proportion of 50% and a 5% margin of error were used. The 50% proportion was selected because no reliable population estimate of the outcome was available, and it produces the maximum required sample under these assumptions. This calculation produced a required sample of approximately 384 participants.

The sample-size calculation was not based on the total general population of the two study areas or on the number of PWE residing in those areas. The number of PWE in the selected communities was unknown. The calculation was therefore used to establish the minimum cross-sectional sample required under the specified precision assumptions.

A total of 650 eligible caregivers were approached: 400 in Mpumalanga and 250 in Limpopo. Of these, 238 from Mpumalanga and 146 from Limpopo participated. The final sample was 384, thereby meeting the calculated minimum sample size requirement. The response rates were 59.5% in Mpumalanga, 58.4% in Limpopo and 59.1% overall.

Although the achieved sample met the calculated target for estimating a proportion, the principal revised analysis used a multivariable continuous-outcome regression model. With 384 observations and approximately 12 model parameters, the model had about 32 observations per estimated parameter. Nevertheless, the study was not prospectively powered for individual regression coefficients, and small associations may have remained undetected.

### 2.6 Sampling and recruitment

The study sites were selected purposively. Eligible caregivers were subsequently identified with assistance from local health and community structures. Potential participants were provided with information about the study and invited to participate voluntarily.

A total of 650 eligible caregivers were approached. Of these, 266 did not participate or did not complete enrolment, resulting in a final sample of 384. Detailed sociodemographic information was not collected from non-participants. Differences between participants and non-participants could therefore not be assessed, and non-response bias remains possible.

### 2.7 Instrument development

Data were collected using a structured, self-developed questionnaire. The instrument was developed after reviewing the study objectives and the literature on epilepsy-related stigma, family caregiving, caregiver burden, access to epilepsy services, cultural beliefs and community support.

The questionnaire contained sections addressing:

- caregiver sociodemographic characteristics;
- caregiving relationships and duration;
- epilepsy-related beliefs and stigma;
- practical, social and financial caregiving burden;
- emotional and safety-related burden;
- health-system and community barriers; and
- perceived epilepsy-related support and enabling conditions.

Agreement items were rated on a five-point Likert scale ranging from 1, “strongly disagree”, to 5, “strongly agree.” Higher scores indicated stronger endorsement of the relevant item or construct. The complete English questionnaire, including the instructions, response options and original item order, is provided in Supplementary File 1.

### 2.8 Pilot testing, language and translation

The questionnaire was pilot-tested among 75 caregivers who did not participate in the main study. The pilot assessment examined the clarity, cultural appropriateness, comprehensibility, administration time and item sequencing. Feedback from the pilot study informed the wording and organization of the final questionnaire.

The questionnaire was administered in English, Xitsonga, Sepedi or isiSwati, according to the participant's language preference. Forward translation from English into each local language and independent back translation into English were undertaken. Differences between the original and back-translated versions were reviewed and reconciled before data collection. The reconciled Xitsonga, Sepedi and isiSwati questionnaire versions administered during data collection are provided in Supplementary File 2.

### 2.9 Measurement of the study constructs

The questionnaire assessed distinct dimensions of caregivers' experiences and perceptions, including practical, social and financial burden; emotional and safety-related burden; health-system and community barriers; and perceived epilepsy-related support and enabling conditions. Construction of the subscales was informed by conceptual coherence (See Supplementary Table S1) and exploratory psychometric assessment. Items that did not demonstrate adequate coherence within a subscale were analyzed individually.

#### 2.9.1 Practical, social and financial burden

This three-item score assessed disruption of daily life, social isolation and financial burden associated with caregiving. Scores ranged from 3 to 15, with higher scores indicating greater practical, social and financial burden.

#### 2.9.2 Emotional and safety-related burden

This two-item score assessed emotional strain and concerns about the PWE's safety. Scores ranged from 2 to 10, with higher scores indicating greater emotional and safety-related burden.

#### 2.9.3 Health-system and community barriers

This four-item score assessed perceived difficulty accessing appropriate services, inadequate community awareness, cultural beliefs and fear of epilepsy within the community. Scores ranged from 4 to 20, with higher scores indicating stronger perceptions of health-system and community barriers.

#### 2.9.4 Perceived epilepsy-related support and enabling environment

The principal outcome was a four-item score assessing caregivers' perceptions of the availability of:

- support groups for caregivers;
- support from community leaders;
- sufficient information to support PWE; and
- Community involvement in reducing epilepsy-related stigma.

The item scores were summed to obtain a continuous score ranging from 4 to 20. Higher scores indicated a more supportive perceived environment for people with epilepsy. This outcome measures perceived contextual support and enabling conditions.

#### 2.9.5 Standalone items

Items concerning epilepsy as a medical condition, witchcraft beliefs, avoidance of PWE, contagion beliefs and the perceived effect of health education were reported individually. They were not combined into a stigma or acceptability scale because they did not demonstrate adequate internal coherence as a single construct.

### 2.10 Psychometric assessment

The ordinal Likert-scale items underwent exploratory psychometric assessment prior to the construction of study scores. Polychoric correlation matrices were used because the response categories were ordered rather than continuous. Overall sampling adequacy was assessed using the Kaiser–Meyer–Olkin statistic, and Bartlett's test of sphericity was used to determine whether the item-correlation matrix was suitable for dimensional assessment. Eigenvalues were examined as preliminary evidence of dimensionality.

The proposed subscales were assessed separately using one-factor principal-axis factoring of their polychoric correlation matrices. Rotation was not applied because one factor was specified for each subscale. Factor loadings, Spearman corrected item-total correlations, conventional Cronbach's alpha, ordinal alpha and McDonald's omega were examined. The analyses were exploratory and were used to assess the internal coherence of the study scores rather than to establish definitive construct validation. Complete item-level psychometric results are presented in Supplementary Table S2.

Because the response options were ordinal, ordinal alpha and McDonald's omega were considered alongside conventional Cronbach's alpha. The internal consistency estimates for the subscales are presented in Table 1. Item-level results and the complete exploratory psychometric output are provided in Supplementary Table S2.

**Table 1:** Internal consistency of the subscales.

Subscale	Number of items	Cronbach's alpha	Ordinal alpha	McDonald's omega
Practical, social and financial burden	3	0.518	0.640	0.645
Emotional and safety-related burden	2	0.808	0.867	0.867
Health-system and community barriers	4	0.618	0.734	0.745
Support and enabling environment	4	0.569	0.723	0.729

The emotional and safety-related burden score demonstrated strong reliability. The ordinal reliability estimates for the barriers and support scores were acceptable for exploratory research. The practical, social and financial burden score showed modest reliability and should be interpreted cautiously. The dimensionality and internal consistency of the Likert-scale items were assessed before constructing the final subscales. Sampling adequacy was examined using the Kaiser–Meyer–Olkin statistic, while Bartlett's test of sphericity was used to evaluate whether the correlation matrix was suitable for dimensional assessment. Complete item-level descriptive and psychometric findings are presented in Supplementary Table S2.

### 2.11 Data collection

Trained data collectors administered the questionnaire in the participants' preferred language. Data collection was conducted in a private setting. Participation was voluntary, and respondents could decline to answer a question or withdraw without penalty.

### 2.12 Data analysis

Data management and descriptive analyses were conducted using IBM SPSS Statistics version 30. Ordinal psychometric analyses, including polychoric correlations, ordinal alpha, principal-axis factor loadings, McDonald's omega and robust regression estimates, were conducted using Python version [insert version] with NumPy, pandas and SciPy. Data completeness, score distributions and observed score ranges were examined before inferential analysis.

Continuous variables were summarized using means and SDs and, where relevant, medians, interquartile ranges and observed ranges. Categorical variables were summarized using frequencies and percentages. Likert items were reported individually by response frequency and combined into subscale scores when supported by the psychometric assessment.

The support and enabling-environment score was analyzed as a continuous outcome. Robust multiple linear regression was used to examine its association with province or study setting, age, sex, marital status, education, employment, relationship to the PWE, practical burden, emotional and safety-related burden and perceived health-system and community barriers. Regression coefficients, 95% Cis and p-values were reported. Statistical significance was assessed at *p* < 0.05.

The original analysis dichotomised the five-item facilitator score using the sample median. This analysis was retained only as a supplementary sensitivity analysis because median dichotomisation discards information and produces a sample-relative category that should not be interpreted as prevalence.

A sensitivity analysis was conducted on the final continuous-outcome regression model without the health-system and community-barrier scores. This analysis examined whether the associations with study location and caregiving burden were dependent on the inclusion of the barrier score.

### 2.13 Ethical considerations

Ethical approval was obtained from the University of Venda Human and Clinical Trials Research Ethics Committee (HCTREC) under Ethical Clearance Number FHS/25/PH/22/1811. Permission to conduct the study was also obtained from the relevant provincial and local authorities. All participants received information about the study's purpose and procedures and provided written informed consent before participating. Confidentiality and anonymity were maintained throughout data collection, analysis and reporting.

## 3 Results

### 3.1 Psychometric performance of the instrument

The overall 20-item correlation matrix demonstrated adequate sampling suitability (Kaiser–Meyer–Olkin = 0.830), and Bartlett's test of sphericity was statistically significant, χ^2^ (190) = 3,296.61, *p* < 0.001. The first six eigenvalues were 6.08, 2.04, 1.82, 1.33, 1.11 and 1.02, indicating that the instrument contained several related but distinguishable dimensions.

The emotional and safety-related burden subscale demonstrated strong internal consistency (ordinal α = 0.867; ω = 0.867). The health-system and community-barrier subscale (ordinal α = 0.734; ω = 0.745) and the support and enabling-environment subscale (ordinal α = 0.723; ω = 0.729) demonstrated acceptable preliminary ordinal reliability. The practical, social and financial burden subscale showed more modest internal consistency (ordinal α = 0.640; ω = 0.645) and was interpreted cautiously. Factor loadings and corrected item-total correlations for all subscales are presented in Supplementary Table S2.

### 3.2 Recruitment and response rate

A total of 650 eligible caregivers were approached, including 400 in Mpumalanga and 250 in Limpopo. Of these, 384 participated: 238 from Mpumalanga and 146 from Limpopo. The response rate was 59.5% in Mpumalanga, 58.4% in Limpopo and 59.1% overall. The required sample of 384 was therefore reached.

### 3.3 Participant characteristics

The caregivers had a mean age of 49.4 years (SD: 14.6), a median age of 49 years and an interquartile range of 39–60 years. Their ages ranged from 18 to 86 years. Parents constituted 47.7% of the participating caregivers. Most participants had provided support to a PWE for more than 3 years (370/384; 96.4%). Twelve participants had provided care for between 1 and 3 years, while two had provided care for between 6 months and 1 year. The complete sociodemographic and caregiving characteristics of the participants are presented in Table 2.

**Table 2:** Sociodemographic and caregiving characteristics of participating caregivers (*N* = 384).

Variable	Category	*n* (%)
Province	Limpopo	146 (38.0)
	Mpumalanga	238 (62.0)
Gender	Male	130 (33.9)
	Female	254 (66.1)
Marital status	Single	186 (48.4)
	Married	77 (20.1)
	Widowed	95 (24.7)
	Divorced	26 (6.8)
Education level	No formal education	88 (22.9)
	Primary	105 (27.3)
	Secondary	134 (34.9)
	Tertiary	57 (14.8)
Employment status	Employed	29 (7.6)
	Unemployed	266 (69.3)
	Self-employed	69 (18.0)
	Student	20 (5.2)
Relationship to PWE	Parent	183 (47.7)
	Sibling	142 (37.0)
	Spouse	34 (8.9)
	Other	25 (6.5)

### 3.4 Epilepsy-related beliefs and stigma

Responses to the standalone epilepsy-related belief and stigma items are presented in Table 3. Most caregivers recognized epilepsy as a medical condition, with 90.9% agreeing or strongly agreeing with this statement. Nevertheless, culturally embedded misconceptions remained evident: 44.8% agreed or strongly agreed that their community believed epilepsy was a form of witchcraft, and 55.7% reported that community members believed epilepsy was contagious.

**Table 3:** Caregivers' responses to standalone epilepsy-related belief and stigma items (*N* = 384).

Item	Strongly disagree, *n* (%)	Disagree, *n* (%)	Neutral, *n* (%)	Agree, *n* (%)	Strongly agree, *n* (%)	Agree/strongly agree, *n* (%)
I feel embarrassed when people know my family member has epilepsy	210 (54.7)	49 (12.8)	22 (5.7)	22 (5.7)	81 (21.1)	103 (26.8)
PWE are treated unfairly in my community	179 (46.6)	73 (19.0)	50 (13.0)	29 (7.6)	53 (13.8)	82 (21.4)
I avoid discussing epilepsy with others to prevent judgment	117 (30.5)	56 (14.6)	48 (12.5)	41 (10.7)	122 (31.8)	163 (42.4)
My community believes epilepsy is a form of witchcraft	66 (17.2)	68 (17.7)	78 (20.3)	56 (14.6)	116 (30.2)	172 (44.8)
I believe epilepsy is a medical condition like any other	18 (4.7)	8 (2.1)	9 (2.3)	50 (13.0)	299 (77.9)	349 (90.9)
People in my community believe epilepsy is contagious	88 (22.9)	48 (12.5)	34 (8.9)	31 (8.1)	183 (47.7)	214 (55.7)
Health education has improved how epilepsy is viewed in my community	72 (18.8)	52 (13.5)	22 (5.7)	23 (6.0)	215 (56.0)	238 (62.0)

These items were analyzed individually because they did not demonstrate sufficient internal consistency to be combined into a single stigma or acceptability subscale. PWE, people with epilepsy.

At the individual or family level, 26.8% felt embarrassed when others knew that their family member had epilepsy, while 42.4% avoided discussing epilepsy to prevent judgment. A further 21.4% perceived that PWE were treated unfairly in their communities. These findings indicate the coexistence of biomedical understanding with persistent epilepsy-related misconceptions, anticipated judgment and discriminatory community attitudes.

### 3.5 Practical, social and financial caregiving burden

Table 4 presents responses to the practical, social and financial burden items. Financial strain was the most frequently endorsed item in this subscale, with 43.0% of caregivers agreeing or strongly agreeing that caregiving had resulted in financial strain. A further 38.8% reported that caregiving affected their ability to work or earn income, while 34.1% reported that it affected their social lives.

**Table 4:** Practical, social and financial caregiving burden (*N* = 384).

Item	Strongly disagree, *n* (%)	Disagree, *n* (%)	Neutral, *n* (%)	Agree, *n* (%)	Strongly agree, *n* (%)	Agree/strongly agree, *n* (%)
Providing care affects my ability to work or earn an income	150 (39.1)	41 (10.7)	44 (11.5)	32 (8.3)	117 (30.5)	149 (38.8)
Caring for someone with epilepsy has affected my social life	139 (36.2)	70 (18.2)	44 (11.5)	33 (8.6)	98 (25.5)	131 (34.1)
I experience financial strain because of my caregiving responsibilities	161 (41.9)	27 (7.0)	31 (8.1)	43 (11.2)	122 (31.8)	165 (43.0)

Higher item scores indicate greater practical, social or financial caregiving burden.

The responses indicate that a considerable proportion of caregivers experienced economic or social disruption. However, the subscale demonstrated modest internal consistency, and its combined score should therefore be interpreted cautiously.

### 3.6 Emotional and safety-related caregiving burden

Emotional and safety-related burden was commonly reported. As shown in Table 5, 66.7% of caregivers agreed or strongly agreed that they felt emotionally drained by their caregiving responsibilities. Almost three-quarters of caregivers (74.2%) agreed or strongly agreed that they frequently worried about the PWE's safety.

**Table 5:** Emotional and safety-related caregiving burden (*N* = 384).

Item	Strongly disagree, *n* (%)	Disagree, *n* (%)	Neutral, *n* (%)	Agree, *n* (%)	Strongly agree, *n* (%)	Agree/strongly agree, *n* (%)
I feel emotionally drained by the responsibility of caregiving	24 (6.2)	54 (14.1)	50 (13.0)	27 (7.0)	229 (59.6)	256 (66.7)
I often worry about the safety of the person with epilepsy	18 (4.7)	34 (8.9)	47 (12.2)	38 (9.9)	247 (64.3)	285 (74.2)

Higher item scores indicate greater emotional and safety-related burden.

Strong agreement was particularly common for both items, suggesting that emotional strain and seizure-related safety concerns were prominent aspects of the caregivers' experiences.

### 3.7 Health-system and community barriers

Caregivers' perceptions of health-system and community barriers are presented in Table 6. Overall, 21.4% agreed or strongly agreed that epilepsy-related health services were difficult to access, while 22.1% reported inadequate epilepsy awareness in schools and workplaces. Cultural beliefs were perceived to interfere with the acceptance of epilepsy by 17.2% of caregivers, while 28.9% reported that community members feared interacting with PWE.

**Table 6:** Perceived health-system and community barriers (*N* = 384).

Item	Strongly disagree, *n* (%)	Disagree, *n* (%)	Neutral, *n* (%)	Agree, *n* (%)	Strongly agree, *n* (%)	Agree/strongly agree, *n* (%)
Health services for epilepsy are difficult to access	197 (51.3)	70 (18.2)	35 (9.1)	60 (15.6)	22 (5.7)	82 (21.4)
There is a lack of epilepsy awareness in schools and workplaces	181 (47.1)	52 (13.5)	66 (17.2)	29 (7.6)	56 (14.6)	85 (22.1)
Cultural beliefs interfere with the acceptance of epilepsy	190 (49.5)	71 (18.5)	57 (14.8)	30 (7.8)	36 (9.4)	66 (17.2)
Community members fear interacting with PWE	209 (54.4)	38 (9.9)	26 (6.8)	55 (14.3)	56 (14.6)	111 (28.9)

The contagion-belief item was treated as a standalone belief item and is presented in Table 3. Higher scores indicate stronger perceptions of health-system and community barriers. PWE, people with epilepsy.

Although fewer than one-third of the caregivers endorsed each revised barrier item, the pattern indicates the continuing presence of service-access difficulties, limited institutional awareness, culturally influenced beliefs and fear of interacting with PWE.

### 3.8 Perceived epilepsy-related support and enabling conditions

Table 7 presents the four items included in the principal support and enabling-environment outcome. Only 7.3% of caregivers agreed or strongly agreed that caregiver support groups were available and helpful. Similarly, 5.7% reported that religious or community leaders promoted the inclusion of PWE, and 7.0% reported having access to information about epilepsy management. Community involvement in reducing epilepsy-related stigma was reported by 11.7% of caregivers.

**Table 7:** Perceived epilepsy-related support and enabling conditions (*N* = 384).

Item	Strongly disagree, *n* (%)	Disagree, *n* (%)	Neutral, *n* (%)	Agree, *n* (%)	Strongly agree, *n* (%)	Agree/strongly agree, *n* (%)
Support groups for caregivers are available and helpful	242 (63.0)	71 (18.5)	43 (11.2)	20 (5.2)	8 (2.1)	28 (7.3)
Religious or community leaders promote the inclusion of PWE	257 (66.9)	67 (17.4)	38 (9.9)	12 (3.1)	10 (2.6)	22 (5.7)
I have access to information about epilepsy management	252 (65.6)	71 (18.5)	34 (8.9)	18 (4.7)	9 (2.3)	27 (7.0)
Community involvement helps to reduce stigma against PWE	260 (67.7)	47 (12.2)	32 (8.3)	21 (5.5)	24 (6.2)	45 (11.7)

Higher item scores indicate a more supportive perceived epilepsy-related environment. The health-education item was analyzed separately because it did not form part of the revised four-item subscale. PWE, people with epilepsy.

Strong disagreement was the most frequent response to all four items. These findings demonstrate that caregivers generally perceived the concrete support and enabling structures measured by this subscale to be poorly available in their communities.

### 3.9 Distribution of subscale scores

The distributions and internal consistency estimates of the revised subscales are presented in Table 8. Emotional and safety-related burden had the highest mean relative to its possible score range. By contrast, the support and enabling-environment score had a mean of 6.52 on a possible range of 4–20, consistent with the low endorsement of the four individual support items.

**Table 8:** Distribution and internal consistency of the subscale scores.

Revised subscale	Items	Possible range	Observed range	Mean (SD)	Median	Cronbach's alpha	Ordinal alpha	McDonald's omega
Practical, social and financial burden	3	3–15	3–15	8.33 (3.65)	8	0.518	0.640	0.645
Emotional and safety-related burden	2	2–10	2–10	8.20 (2.37)	10	0.808	0.867	0.867
Health-system and community barriers	4	4–20	4–20	8.69 (3.90)	8	0.618	0.734	0.745
Support and enabling environment	4	4–20	4–17	6.52 (2.77)	6	0.569	0.723	0.729

Higher scores represent greater endorsement of the construct named in each row. Reliability estimates are preliminary because the instrument was self-developed and the subscales contain relatively few items.

### 3.10 Factors associated with perceived support and enabling environments

As shown in Table 9, the robust multiple linear regression model explained 27.7% of the variation in perceived support and enabling-environment scores (*R*^2^ = 0.277; adjusted *R*^2^ = 0.254).

**Table 9:** Robust multiple linear regression of factors associated with the support and enabling-environment score.

Variable	Adjusted coefficient (B)	95% CI	*p*-value
Mpumalanga vs. Limpopo	−1.13	−1.71 to −0.54	< 0.001
Age, per year	−0.010	−0.032 to 0.012	0.377
Female vs. male	0.37	−0.17 to 0.91	0.175
Married vs. never married	0.10	−0.63 to 0.82	0.794
Widowed vs. never married	0.26	−0.37 to 0.89	0.412
Divorced vs. never married	−0.27	−1.25 to 0.71	0.588
Secondary/tertiary vs. lower education	0.17	−0.49 to 0.83	0.618
Unemployed/student vs. employed	0.06	−0.65 to 0.76	0.878
Non-parent caregiver vs. parent	0.22	−0.32 to 0.75	0.428
Practical, social and financial burden	−0.04	−0.12 to 0.04	0.289
Emotional and safety-related burden	−0.31	−0.44 to −0.18	< 0.001
Health-system and community barriers	0.30	0.22 to 0.38	< 0.001

Caregivers from the selected Mpumalanga sites had support and enabling-environment scores that were, on average, 1.13 points lower than those of caregivers from the selected Limpopo sites. A greater emotional and safety-related burden was associated with lower perceived support. Practical, social and financial burden were not independently associated with the outcome.

The positive coefficient for health-system and community barriers indicates that caregivers who reported more barriers also tended to report greater availability of some enabling conditions. This result should not be interpreted as evidence that barriers improve support. It may reflect greater engagement with epilepsy-related services, increased awareness of both barriers and supports, common method variance or a general tendency to endorse similarly structured questionnaire statements.

Age was not independently associated with the revised continuous outcome after adjustment for education, employment and the other covariates. The results, therefore, do not provide evidence for a generational explanation of caregivers' perceptions.

### 3.11 Sensitivity analysis excluding health-system and community barriers

The continuous-outcome regression model was repeated without the health-system and community-barrier score. Emotional and safety-related burden remained negatively associated with perceived support and enabling-environment scores [B = −0.48; 95% CI (−0.61, −0.35); *p* < 0.001]. The difference between the selected study sites also remained evident, with caregivers at the Mpumalanga site reporting lower scores than those at the Limpopo site [B = −1.44; 95% CI (−2.12, −0.75); *p* < 0.001].

Practical, social and financial burden remained unassociated with the outcome [B = 0.05; 95% CI (−0.03, 0.13); *p* = 0.233], and age remained statistically non-significant [B = −0.016; 95% CI (−0.040, 0.007); *p* = 0.176]. The sensitivity model explained 13.5% of the variation in support and enabling-environment scores. These findings indicate that the association between emotional and safety-related burden and perceived support did not depend on whether the barrier score was included.

## 4 Discussion

This study found that caregivers perceived limited epilepsy-related support and enabling conditions in the selected rural communities. Only 7.3% reported that helpful caregiver support groups were available, 5.7% indicated that religious or community leaders promoted the inclusion of people with epilepsy (PWE), 7.0% had access to epilepsy-management information, and 11.7% perceived community involvement in stigma reduction. The low support and enabling-environment score, therefore, reflects limited access to concrete community and informational resources. These findings are concerning because caregivers require accessible information, psychosocial assistance and supportive community structures to manage epilepsy-related responsibilities effectively. Previous evidence similarly demonstrates that caregivers frequently have unmet informational and social-support needs (5).

Caregivers at the selected Mpumalanga sites reported lower support and enabling-environment scores than those at the Limpopo sites. This difference may reflect variations in local health services, community programmes, leadership involvement, information provision or referral arrangements. Previous research in rural Limpopo and Mpumalanga identified gaps in caregivers' epilepsy-related knowledge and practices, while healthcare providers described limitations in the support available to caregivers and family members (9). Research on healthcare access in resource-constrained settings has similarly demonstrated that geographical distance, limited specialist services, transportation difficulties, costs and insufficient information may influence families' access to neurological care (10, 11).

Emotional and safety-related burden was negatively associated with perceived support. This finding is supported by the high proportions of caregivers who felt emotionally drained (66.7%) or worried about the PWE's safety (74.2%). Epilepsy caregiving may involve persistent vigilance because seizures are often unpredictable and may result in injury or require emergency assistance. This can contribute to anxiety, uncertainty and emotional exhaustion. Similar experiences have been reported among caregivers of both children and adults with epilepsy (5, 12). The cross-sectional design prevents determining whether inadequate support increased burden or whether caregivers experiencing greater burden perceived their environment as less supportive. Nevertheless, the findings demonstrate the need to include caregiver emotional wellbeing, seizure first aid and safety planning within epilepsy services.

Although many caregivers perceived health education as having improved community views of epilepsy, concrete support structures remained poorly available. Few caregivers reported access to helpful support groups, epilepsy-management information, supportive community leadership or community involvement in stigma reduction. The findings suggest that awareness activities may be recognized and valued without necessarily developing into sustained systems of practical and psychosocial support. Systematic syntheses of caregivers' experiences indicate that families require support from healthcare professionals, relatives, peers and wider community structures (5, 13). In rural and resource-constrained settings, limited access to information and formal support may leave families responsible for managing complex seizure-related and psychosocial needs with insufficient assistance (10, 14). The limited support reported in the present study is therefore consistent with broader evidence that epilepsy care must address social and informational needs alongside clinical treatment.

Community programmes should progress beyond isolated awareness campaigns by establishing referral pathways, locally accessible epilepsy information, caregiver support groups and partnerships with community and traditional leadership. Such multilevel action is consistent with the WHO global action plan, which calls for coordinated responses involving health services, communities, people with epilepsy and their families (7).

Health-system and community barriers were positively associated with perceived support and enabling conditions. This unexpected association should not be interpreted as indicating that barriers improve support. Caregivers who interacted more frequently with health and community systems may have become more aware of both available resources and system deficiencies. The relationship may also reflect common-method variance or acquiescence because both constructs were measured through self-reported Likert-scale items (15, 16). Qualitative and longitudinal studies are required to determine whether this association reflects service engagement, heightened awareness or measurement effects.

Although 90.9% recognized epilepsy as a medical condition, 44.8% reported community beliefs associating epilepsy with witchcraft, 55.7% reported beliefs that epilepsy was contagious, and 42.4% avoided discussing epilepsy to prevent judgment. Biomedical understanding, therefore, coexisted with cultural misconceptions and anticipated stigma. Such beliefs remain common in sub-Saharan Africa and may promote fear, social distancing and delayed use of biomedical care (2). Although 62.0% believed that health education had improved community perceptions, education alone had not translated into widely available support structures.

These findings support culturally responsive education that directly addresses witchcraft, contagion and seizure management, together with caregiver support groups, accessible information and psychosocial services. Community, religious and traditional leaders should be engaged alongside PWE, caregivers and healthcare workers. The findings should nevertheless be interpreted in view of purposive site selection, a 59.1% response rate, possible non-response bias, the absence of clinical and functional-dependency information, self-reported measurement and the cross-sectional design. Further validation of the study instrument and longitudinal investigation of caregiver burden, service access and community support are warranted.

## 5 Implications for practice

Health services should provide caregivers with clear, culturally and linguistically appropriate information about seizure management, treatment adherence, injury prevention and referral pathways. Because caregiver burden may include anxiety, emotional exhaustion and continuing concern about seizure-related injuries, routine epilepsy care should also include assessment of caregiver wellbeing and referral for psychosocial support where required (5, 12).

Community-based caregiver groups could provide opportunities for shared learning, emotional support and reduced social isolation. Evidence from caregiver syntheses indicates that support should be derived from multiple sources, including healthcare professionals, family networks, peers and community structures (13). Community, religious and traditional leaders should therefore participate in stigma-reduction programmes alongside healthcare workers, people with epilepsy and caregivers.

Interventions should directly address beliefs concerning witchcraft and contagion while recognizing that such beliefs are situated within local cultural and social systems (17, 18). This approach is consistent with international recommendations for intersectoral epilepsy responses that integrate healthcare, social support, awareness, human rights and the meaningful participation of people with epilepsy and their families (7).

## 6 Strengths and limitations

This study contributes evidence on caregivers' perceptions of epilepsy-related support and enabling conditions in two rural South African settings. The calculated sample size of 384 caregivers was achieved, with only one key caregiver representing each PWE and household. The questionnaire was administered in participants' preferred languages following forward and back translation and was pilot-tested among 75 caregivers who were not included in the main study. The use of a continuous primary outcome retained the information contained in the support score, while the psychometric assessment enabled stigma-related experiences, caregiving burden, barriers and contextual support to be examined as distinct domains.

Several limitations should be considered. The study sites were purposively selected; therefore, the findings cannot be generalized to all caregivers in Limpopo, Mpumalanga or South Africa. The number of PWE residing in the selected municipalities was not known, and individual sampling probabilities and population coverage could not be calculated. The overall response rate was 59.1%, and sociodemographic information was unavailable for non-participants, raising the possibility of non-response bias. The study did not assess epilepsy type, seizure frequency, severity, treatment status or functional dependence. It was therefore not possible to determine whether caregivers' experiences differed by the clinical condition or the level of support required by the PWE. The questionnaire was self-developed, and some subscales contained few items or demonstrated modest internal consistency. The psychometric results should consequently be regarded as preliminary. All measures were self-reported and collected using similar Likert-scale response formats. Social desirability bias, acquiescence and common method variance may have influenced the results, particularly the positive association between perceived barriers and support. Finally, the cross-sectional design does not establish causal or temporal relationships between caregiving burden, barriers and perceived support.

## 7 Future directions

Future studies should use probability-based sampling when reliable epilepsy registers or community enumeration systems are available. Data on epilepsy type, seizure frequency, clinical severity, treatment status, functional independence and the level of assistance required by the PWE should be collected to determine how these factors influence caregiver experiences.

The instrument requires further development through cognitive interviewing, independent content validity assessment, confirmatory factor analysis, test–retest reliability, and measurement invariance testing across languages and study settings. Direct family acceptance should be measured separately using indicators of family attitudes, inclusion, respect, decision-making and behavior toward PWE.

Longitudinal and mixed-methods studies are needed to clarify the relationships among emotional burden, service access, community barriers and available support. Intervention studies should evaluate whether culturally responsive epilepsy education, caregiver support groups, accessible management information and sustained involvement of community, religious and traditional leaders improve caregiver wellbeing and the social inclusion of PWE.

## 8 Conclusion

Caregivers in the selected rural study sites perceived limited availability of caregiver support groups, epilepsy-management information, community leadership support and organized community participation in stigma reduction. Lower support and enabling-environment scores were observed at the selected Mpumalanga site and among caregivers experiencing greater emotional and safety-related burden. These associations remained evident when the health-system and community-barrier score was excluded from the model.

Biomedical recognition of epilepsy coexisted with beliefs concerning witchcraft and contagion and with avoidance of epilepsy-related disclosure. These findings support culturally responsive epilepsy education, caregiver psychosocial support, accessible seizure-management information and sustained engagement with community, religious and traditional leaders. The findings describe the selected study settings and should not be interpreted as representative of Limpopo, Mpumalanga or South Africa as a whole.

## Data Availability

The raw data supporting the conclusions of this article will be made available by the authors, without undue reservation.

## References

[1] WHO. Epilepsy (2024). Aavailable online at: https://www.who.int/news-room/fact-sheets/detail/epilepsy (Accessed May 2, 2026).

[2] Kaddumukasa M Kaddumukasa MN Buwembo W Munabi IG Blixen C Lhatoo S . Epilepsy misconceptions and stigma reduction interventions in sub-Saharan Africa, a systematic review. Epilepsy Behav. (2018) 85:21–7. doi: 10.1016/j.yebeh.2018.04.014 PMC6355646 29906697

[3] Austin JK Birbeck G Parko K Kwon CS Fernandes PT Braga P . Epilepsy-related stigma and attitudes: systematic review of screening instruments and interventions—Report by the international league against epilepsy task force on stigma in epilepsy. Epilepsia. (2022) 63:598–628. doi: 10.1111/epi.17133 34985766

[4] Boele F Jensen C Johnson GM Lammers-Spijker A Altinbas A van den Berg L . Health-related quality of life and unmet needs of people with epilepsy and their family caregivers: a systematic scoping review. Epilepsy Behav. (2025) 171:110601. doi: 10.1016/j.yebeh.2025.110601 40712205

[5] Yu Z Shao Q Hou K Wang Y Sun X. The experiences of caregivers of children with epilepsy: a meta-synthesis of qualitative research studies. Front. Psychiatry. (2022) 13:987892. doi: 10.3389/fpsyt.2022.987892 PMC9513543 36177220

[6] Seid S Demilew D Yimer S Mihretu A. Prevalence and associated factors of mental distress among caregivers of patients with epilepsy in ethiopia: a cross-sectional study design. Psychiatry J. (2018) 2018:1–8. doi: 10.1155/2018/2819643 PMC6180922 30363640

[7] WHO. Intersectoral Global Action Plan on Epilepsy and Other Neurological Disorders 2022–2031. Geneva: World Health Organization (2023).

[8] Musekwa OP Makhado L Maphula A. Caregivers' and family members' knowledge attitudes and practices (KAP) toward epilepsy in rural Limpopo and Mpumalanga, South Africa. Int J Environ Res Public Health. (2023) 20:5222. doi: 10.3390/ijerph20065222 PMC10048962 36982132

[9] Musekwa OP Makhado L. It goes beyond anxiety: experiences of family members and caregivers of epilepsy care and support. Neuropsychiatr Dis Treat. (2023) 19:2757–64. doi: 10.2147/NDT.S430337 PMC10712244 38090018

[10] Makhado L Maphula A Ngomba RT Musekwa OP Makhado TG Nemathaga M . Epilepsy in rural South Africa: patient experiences and healthcare challenges. Epilepsia Open. (2024) 9:1565–74. doi: 10.1002/epi4.12999 PMC11296125 38884148

[11] Mwangi LW Abuga JA Cottrell E Kariuki SM Kinyanjui SM Newton CR. Barriers to access and utilization of healthcare by children with neurological impairments and disability in low-and middle-income countries: a systematic review. Wellcome Open Res. (2022) 6:61. doi: 10.12688/wellcomeopenres.16593.2 PMC8902259 35299711

[12] Yeni K Tulek Z Cavusoglu A Dunya CP Erden SO Bostan NS . Caregiver burden and its predictors in adult epilepsy patients. Epilepsy Behav. (2024) 153:109685. doi: 10.1016/j.yebeh.2024.109685 38368790

[13] Yang L Ji J Lu Q Tang P Jiang Y Yang H . Caregivers' experiences in the management of children with epilepsy: a systematic synthesis of qualitative studies. Seizure. (2023) 106:117–28. doi: 10.1016/j.seizure.2023.02.004 36827863

[14] Okiah L Olowo S Iramiot SJ Nekaka R Ssenyonga LVN. Lived experiences of caregivers of persons with epilepsy attending an epilepsy clinic at a tertiary hospital, eastern Uganda: a phenomenological approach. PLoS ONE. (2023) 18:e0274373. doi: 10.1371/journal.pone.0274373 PMC10353802 37463142

[15] Podsakoff PM MacKenzie SB Lee J-Y Podsakoff NP. Common method biases in behavioral research: a critical review of the literature and recommended remedies. J Appl Psychol. (2003) 88:879–903. doi: 10.1037/0021-9010.88.5.879 14516251

[16] Suárez-Alvarez J Pedrosa I Lozano LM García-Cueto E Cuesta M Muñiz J. El uso de ítems inversos en las escalas tipo Likert: una práctica cuestionable. Psicothema. (2018) 30:149–58. doi: 10.7334/psicothema2018.33 29694314

[17] Braga P Hosny H Kakooza-Mwesige A Rider F Tripathi M Guekht A. How to understand and address the cultural aspects and consequences of diagnosis of epilepsy, including stigma. Epileptic Disord. (2020) 22:531–47. doi: 10.1684/epd.2020.1201 33079064

[18] Nemathaga M Maputle M Makhado L Mashau N. Views of traditional healers on collaboration with health professionals when managing epilepsy in rural areas of Limpopo and Mpumalanga Provinces (South Africa). Epilepsy paroxysmal cond. (2023) 15:222–31. doi: 10.17749/2077-8333/epi.par.con.2023.165

