# Supplementary material for: Perceived epilepsy-related support and enabling environments and associated caregiver factors in rural South Africa: a cross-sectional study in Limpopo and Mpumalanga
Source: Front Public Health. 2026 Sep 7;14:1881761. doi: 10.3389/fpubh.2026.1881761 (PMC13590295; doi:10.3389/fpubh.2026.1881761)
Supplement: Supplementary file 1 [file Supplementary_File_1.docx]

**Supplementary File 1**

**Instrument title:** Questionnaire: Acceptability of People with Epilepsy

**Section A. Demographic profile**

**A1. Age:** ________ years

**A2. Gender:** ☐ Male	☐ Female	☐ Other

**A3. Marital status:** ☐ Single	☐ Married	☐ Widowed	☐ Divorced

**A4. Education level:** ☐ No formal education	☐ Primary	☐ Secondary	☐ Tertiary

**A5. Employment status:** ☐ Employed	☐ Unemployed	☐ Self-employed	☐ Student

**A6. Relationship to the person with epilepsy:** ☐ Parent	☐ Sibling	☐ Spouse	☐ Other: __________

**A7. Duration of caregiving:** ☐ <6 months	☐ 6 months–1 year	☐ 1–3 years	☐ >3 years

**Response scale for Sections B–E**

1 = Strongly disagree; 2 = Disagree; 3 = Neutral; 4 = Agree; 5 = Strongly agree.

**Section B. Epilepsy-related beliefs and stigma**

| **Item** | **Statement** | **1** | **2** | **3** | **4** | **5** |
| --- | --- | --- | --- | --- | --- | --- |
| S1 | I feel embarrassed when people know my family member has epilepsy. | ☐ | ☐ | ☐ | ☐ | ☐ |
| S2 | People with epilepsy are treated unfairly in my community. | ☐ | ☐ | ☐ | ☐ | ☐ |
| S3 | I avoid discussing epilepsy with others to prevent judgment. | ☐ | ☐ | ☐ | ☐ | ☐ |
| S4 | My community believes epilepsy is a form of witchcraft. | ☐ | ☐ | ☐ | ☐ | ☐ |
| S5 | I believe epilepsy is a medical condition like any other. | ☐ | ☐ | ☐ | ☐ | ☐ |

**Section C. Burden of care**

| **Item** | **Statement** | **1** | **2** | **3** | **4** | **5** |
| --- | --- | --- | --- | --- | --- | --- |
| B1 | Providing care affects my ability to work or earn income. | ☐ | ☐ | ☐ | ☐ | ☐ |
| B2 | I feel emotionally drained by the responsibility of caregiving. | ☐ | ☐ | ☐ | ☐ | ☐ |
| B3 | Caring for someone with epilepsy has affected my social life. | ☐ | ☐ | ☐ | ☐ | ☐ |
| B4 | I often worry about the safety of the person with epilepsy. | ☐ | ☐ | ☐ | ☐ | ☐ |
| B5 | I have financial strain due to caregiving responsibilities. | ☐ | ☐ | ☐ | ☐ | ☐ |

**Section D. Barriers**

| **Item** | **Statement** | **1** | **2** | **3** | **4** | **5** |
| --- | --- | --- | --- | --- | --- | --- |
| D1 | People in my community believe epilepsy is contagious. | ☐ | ☐ | ☐ | ☐ | ☐ |
| D2 | Health services for epilepsy are difficult to access. | ☐ | ☐ | ☐ | ☐ | ☐ |
| D3 | There is a lack of epilepsy awareness in schools and workplaces. | ☐ | ☐ | ☐ | ☐ | ☐ |
| D4 | Cultural beliefs interfere with the acceptance of epilepsy. | ☐ | ☐ | ☐ | ☐ | ☐ |
| D5 | Community members fear interacting with people who have epilepsy. | ☐ | ☐ | ☐ | ☐ | ☐ |

**Section E. Support and enabling conditions**

| **Item** | **Statement** | **1** | **2** | **3** | **4** | **5** |
| --- | --- | --- | --- | --- | --- | --- |
| E1 | Health education has improved how epilepsy is viewed in my community. | ☐ | ☐ | ☐ | ☐ | ☐ |
| E2 | Support groups for caregivers are available and helpful. | ☐ | ☐ | ☐ | ☐ | ☐ |
| E3 | Religious or community leaders promote the inclusion of PWE. | ☐ | ☐ | ☐ | ☐ | ☐ |
| E4 | I have access to information about epilepsy management. | ☐ | ☐ | ☐ | ☐ | ☐ |
| E5 | Community involvement helps reduce stigma against PWE. | ☐ | ☐ | ☐ | ☐ | ☐ |

**Scoring used in the analysis**

All agreement items were coded from 1 to 5 in the direction shown. The final subscale scores were calculated by summing the specified items; no reverse scoring was applied in the revised analysis. Standalone items were reported individually and were not included in a total “acceptability” score.

| **Final score** | **Items** | **Possible range** |
| --- | --- | --- |
| Practical, social and financial burden | B1, B3, B5 | 3–15 |
| Emotional and safety-related burden | B2, B4 | 2–10 |
| Health-system and community barriers | D2, D3, D4, D5 | 4–20 |
| Support and enabling environment | E2, E3, E4, E5 | 4–20 |
