# Supplementary material for: Perceived epilepsy-related support and enabling environments and associated caregiver factors in rural South Africa: a cross-sectional study in Limpopo and Mpumalanga
Source: Front Public Health. 2026 Sep 7;14:1881761. doi: 10.3389/fpubh.2026.1881761 (PMC13590295; doi:10.3389/fpubh.2026.1881761)
Supplement: Supplementary file 2 [file Supplementary_File_2.docx]

**Supplementary File 2**

**Questionnaire translations: Xitsonga, Sepedi and isiSwati**

**XITSONGA**

**XIVUTISO: MAVONELO NA NSHETIKELO MAYELANA NA VANHU LAVA HANYAKA NA VUVABYI BYA KU WA (EPILEPSY)**

Xivutiso lexi xi kongomisiwa eka muhlayisi lonkulu wa munhu loyi a hanyaka na vuvabyi bya ku wa (epilepsy). PWE swi vula vanhu lava hanyaka na vuvabyi bya ku wa.

**XIYENGE A: VUXOKOXOKO BYA MUHLAMURI**

**A1. Malembe: ________**

**A2. Rimbewu** ☐ Wanuna	☐ Wansati	☐ Rin’wana

**A3. Xiyimo xa vukati** ☐ A nga tekiwanga/teka	☐ U tekile/tekiwile	☐ Noni	☐ U dlaye vukati

**A4. Dyondzo leyi fikeleriweke** ☐ A nga dyondzanga exikolweni	☐ Puraimari	☐ Sekondari	☐ Dyondzo ya le henhla

**A5. Xiyimo xa ntchito** ☐ Wa tirha	☐ A nga tirhi	☐ Wa titirha	☐ Xichudeni

**A6. Vuxaka na munhu loyi a hanyaka na vuvabyi bya ku wa** ☐ Mutswari	☐ Makweru	☐ Nkatakulobye	☐ Swin’wana: __________

**A7. Nkarhi lowu u nga va muhlayisi** ☐ Ehansi ka tin’hweti ta 6	☐ Tin’hweti ta 6–lembe rin’we	☐ Malembe ya 1–3	☐ Ku hundza 3 wa malembe

**MPIMO WA TINHLAMULO / SEKALA SA DIKARABO / SIKALI SETIMPHENDVULO**

Eka swivulwa leswi landzelaka, komba mpimo lowu u pfumelelanaka na swona. Hlawula nhlamulo yin’we eka xivulwa xin’wana na xin’wana.

1 = A ndzi pfumelelani na swona nikatsongo

2 = A ndzi pfumelelani

3 = A ndzi na vonelo

4 = Ndza pfumela

5 = Ndza pfumela swinene

**XIYENGE B: MAVONELO NA KU SORIWA LOKU FAMBELANAKA NA VUVABYI BYA KU WA**

| **Nomboro** | **Xivulwa** | **1** | **2** | **3** | **4** | **5** |
| --- | --- | --- | --- | --- | --- | --- |
| S1 | Ndza khomiwa hi tingana loko vanhu va tiva leswaku xirho xa ndyangu wa mina xi na vuvabyi bya ku wa. | ☐ | ☐ | ☐ | ☐ | ☐ |
| S2 | Vanhu lava hanyaka na vuvabyi bya ku wa va khomiwa hi ndlela leyi nga lulamangiki emugangeni wa mina. | ☐ | ☐ | ☐ | ☐ | ☐ |
| S3 | Ndza papalata ku burisana ni van’wana hi vuvabyi bya ku wa leswaku ndzi nga avanyisiwi. | ☐ | ☐ | ☐ | ☐ | ☐ |
| S4 | Vanhu va le mugangeni wa mina va tshemba leswaku vuvabyi bya ku wa byi vangiwa hi vuloyi. | ☐ | ☐ | ☐ | ☐ | ☐ |
| S5 | Ndza tshemba leswaku vuvabyi bya ku wa i vuvabyi bya swa vutshunguri ku fana ni mavabyi man’wana. | ☐ | ☐ | ☐ | ☐ | ☐ |

**XIYENGE C: NDZWALO WA VUHLAYISI**

| **Nomboro** | **Xivulwa** | **1** | **2** | **3** | **4** | **5** |
| --- | --- | --- | --- | --- | --- | --- |
| B1 | Ku nyika vuhlayisi swi khumba vuswikoti bya mina byo tirha kumbe ku kuma muholo. | ☐ | ☐ | ☐ | ☐ | ☐ |
| B2 | Ndza karhala emoyeni hikokwalaho ka vutihlamuleri bya ku va muhlayisi. | ☐ | ☐ | ☐ | ☐ | ☐ |
| B3 | Ku hlayisa munhu loyi a hanyaka na vuvabyi bya ku wa swi khumbe vutomi bya mina bya vuxaka ni van’wana. | ☐ | ☐ | ☐ | ☐ | ☐ |
| B4 | Minkarhi yo tala ndzi vilela hi vuhlayiseki bya munhu loyi a hanyaka na vuvabyi bya ku wa. | ☐ | ☐ | ☐ | ☐ | ☐ |
| B5 | Ndza tikeriwe hi timali hikokwalaho ka vutihlamuleri bya vuhlayisi. | ☐ | ☐ | ☐ | ☐ | ☐ |

**XIYENGE D: SWIPHINGO**

| **Nomboro** | **Xivulwa** | **1** | **2** | **3** | **4** | **5** |
| --- | --- | --- | --- | --- | --- | --- |
| D1 | Vanhu va le mugangeni wa mina va tshemba leswaku vuvabyi bya ku wa bya tlulela. | ☐ | ☐ | ☐ | ☐ | ☐ |
| D2 | Swi tika ku fikelela vukorhokeri bya rihanyo bya vuvabyi bya ku wa. | ☐ | ☐ | ☐ | ☐ | ☐ |
| D3 | Ku na ku pfumaleka ka vutivi mayelana na vuvabyi bya ku wa eswikolweni ni le tindhawini ta mintirho. | ☐ | ☐ | ☐ | ☐ | ☐ |
| D4 | Minkhuva ni tidyondzo ta ndhavuko swi kavanyeta ku amukeleka ka vanhu lava hanyaka na vuvabyi bya ku wa. | ☐ | ☐ | ☐ | ☐ | ☐ |
| D5 | Vanhu va le mugangeni va chava ku tirhisana ni vanhu lava hanyaka na vuvabyi bya ku wa. | ☐ | ☐ | ☐ | ☐ | ☐ |

**XIYENGE E: NSHETIKELO NA XIYIMO LEXI PFUNAKA**

| **Nomboro** | **Xivulwa** | **1** | **2** | **3** | **4** | **5** |
| --- | --- | --- | --- | --- | --- | --- |
| E1 | Dyondzo ya rihanyo yi antswise ndlela leyi vuvabyi bya ku wa byi voniwaka ha yona emugangeni wa mina. | ☐ | ☐ | ☐ | ☐ | ☐ |
| E2 | Mintlawa ya nseketelo ya vahlayisi ya kumeka naswona ya pfuna. | ☐ | ☐ | ☐ | ☐ | ☐ |
| E3 | Varhangeri va vukhongeri kumbe va muganga va khutaza leswaku vanhu lava hanyaka na vuvabyi bya ku wa va katsiwa. | ☐ | ☐ | ☐ | ☐ | ☐ |
| E4 | Ndza swi kota ku kuma vuxokoxoko mayelana na malawulelo ya vuvabyi bya ku wa. | ☐ | ☐ | ☐ | ☐ | ☐ |
| E5 | Ku nghenelela ka muganga swi pfuna ku hunguta ku soriwa ka vanhu lava hanyaka na vuvabyi bya ku wa. | ☐ | ☐ | ☐ | ☐ | ☐ |

**SEPEDI**

**POTŠIŠONYAKIŠIŠO: MAIKUTLO LE THEKGO MABAPI LE BATHO BAO BA PHELAGO LE BOLWETŠI BJA GO WA (EPILEPSY)**

Potšišonyakišišo ye e lebane le mohlokomedi yo mogolo wa motho yo a phelago le bolwetši bja go wa (epilepsy). PWE e ra batho bao ba phelago le bolwetši bja go wa.

**KAROLO A: TSEBIŠO YA MOTŠEAKAROLO**

**A1. Mengwaga: ________**

**A2. Bong** ☐ Monna	☐ Mosadi	☐ Bong bjo bongwe

**A3. Maemo a lenyalo** ☐ Ga se a nyala/nyalwa	☐ O nyetše/nyetšwe	☐ Mohlologadi/Mohlolo	☐ O hladilwe

**A4. Maemo a thuto** ☐ Ga a na thuto ya semmušo	☐ Thuto ya fase	☐ Thuto ya magareng	☐ Thuto ya godimo

**A5. Maemo a mošomo** ☐ O a šoma	☐ Ga a šome	☐ O a itšhomela	☐ Moithuti

**A6. Kamano le motho yo a phelago le bolwetši bja go wa** ☐ Motswadi	☐ Ngwanešo	☐ Molekane	☐ Tše dingwe: __________

**A7. Nako ya go ba mohlokomedi** ☐ Ka fase ga dikgwedi tše 6	☐ Dikgwedi tše 6–ngwaga o tee	☐ Mengwaga e 1–3	☐ Go feta mengwaga e 3

**MPIMO WA TINHLAMULO / SEKALA SA DIKARABO / SIKALI SETIMPHENDVULO**

Go dipolelo tše di latelago, bontšha tekanyo yeo o dumelelanago le tšona. Kgetha karabo e tee polelong ye nngwe le ye nngwe.

1 = Ga ke dumele le gatee

2 = Ga ke dumele

3 = Ga ke tšee lehlakore

4 = Ke a dumela

5 = Ke dumela kudu

**KAROLO B: DIKGOPOLO LE KGOBOSO TŠEO DI AMANAGO LE BOLWETŠI BJA GO WA**

| **Nomoro** | **Polelo** | **1** | **2** | **3** | **4** | **5** |
| --- | --- | --- | --- | --- | --- | --- |
| S1 | Ke lewa ke dihlong ge batho ba tseba gore setho sa lapa la ka se na le bolwetši bja go wa. | ☐ | ☐ | ☐ | ☐ | ☐ |
| S2 | Batho bao ba phelago le bolwetši bja go wa ba swarwa ka go hloka toka setšhabeng sa gešo. | ☐ | ☐ | ☐ | ☐ | ☐ |
| S3 | Ke efoga go bolela le batho ba bangwe ka bolwetši bja go wa gore ke se ke ka ahlolwa. | ☐ | ☐ | ☐ | ☐ | ☐ |
| S4 | Batho ba setšhaba sa gešo ba dumela gore bolwetši bja go wa bo bakwa ke boloi. | ☐ | ☐ | ☐ | ☐ | ☐ |
| S5 | Ke dumela gore bolwetši bja go wa ke bolwetši bja kalafo go swana le malwetši a mangwe. | ☐ | ☐ | ☐ | ☐ | ☐ |

**KAROLO C: MORWALO WA TLHOKOMELO**

| **Nomoro** | **Polelo** | **1** | **2** | **3** | **4** | **5** |
| --- | --- | --- | --- | --- | --- | --- |
| B1 | Go fana ka tlhokomelo go ama bokgoni bja ka bja go šoma goba go hwetša letseno. | ☐ | ☐ | ☐ | ☐ | ☐ |
| B2 | Ke ikwa ke lapile maikwelong ka lebaka la maikarabelo a go ba mohlokomedi. | ☐ | ☐ | ☐ | ☐ | ☐ |
| B3 | Go hlokomela motho yo a phelago le bolwetši bja go wa go amile bophelo bja ka bja leago. | ☐ | ☐ | ☐ | ☐ | ☐ |
| B4 | Gantši ke tshwenyega ka polokego ya motho yo a phelago le bolwetši bja go wa. | ☐ | ☐ | ☐ | ☐ | ☐ |
| B5 | Ke na le mathata a ditšhelete ka lebaka la maikarabelo a tlhokomelo. | ☐ | ☐ | ☐ | ☐ | ☐ |

**KAROLO D: MAPHEKO**

| **Nomoro** | **Polelo** | **1** | **2** | **3** | **4** | **5** |
| --- | --- | --- | --- | --- | --- | --- |
| D1 | Batho ba setšhaba sa gešo ba dumela gore bolwetši bja go wa bo a fetela. | ☐ | ☐ | ☐ | ☐ | ☐ |
| D2 | Go thata go fihlelela ditirelo tša maphelo tša bolwetši bja go wa. | ☐ | ☐ | ☐ | ☐ | ☐ |
| D3 | Go na le tlhokego ya temogo ka bolwetši bja go wa dikolong le mafelong a mošomo. | ☐ | ☐ | ☐ | ☐ | ☐ |
| D4 | Ditumelo tša setšo di šitiša kamogelo ya batho bao ba phelago le bolwetši bja go wa. | ☐ | ☐ | ☐ | ☐ | ☐ |
| D5 | Batho ba setšhaba ba boifa go dirišana le batho bao ba phelago le bolwetši bja go wa. | ☐ | ☐ | ☐ | ☐ | ☐ |

**KAROLO E: THEKGO LE MAEMO AO A THUŠAGO**

| **Nomoro** | **Polelo** | **1** | **2** | **3** | **4** | **5** |
| --- | --- | --- | --- | --- | --- | --- |
| E1 | Thuto ya maphelo e kaonafaditše tsela yeo bolwetši bja go wa bo lebelelwago ka yona setšhabeng sa gešo. | ☐ | ☐ | ☐ | ☐ | ☐ |
| E2 | Dihlopha tša thekgo tša bahlokomedi di gona ebile di a thuša. | ☐ | ☐ | ☐ | ☐ | ☐ |
| E3 | Baetapele ba bodumedi goba ba setšhaba ba hlohleletša go akaretšwa ga batho bao ba phelago le bolwetši bja go wa. | ☐ | ☐ | ☐ | ☐ | ☐ |
| E4 | Ke kgona go hwetša tshedimošo mabapi le taolo ya bolwetši bja go wa. | ☐ | ☐ | ☐ | ☐ | ☐ |
| E5 | Go tšea karolo ga setšhaba go thuša go fokotša kgobošo ya batho bao ba phelago le bolwetši bja go wa. | ☐ | ☐ | ☐ | ☐ | ☐ |

**ISISWATI**

**LUHLU LWEMIBUTO: IMIBONO NEKWESEKELWA MAYELANA NEBANTFU LABAPHILA NESIFO SEKUWA (EPILEPSY)**

Loluhlu lwemibuto lubhekiswe kumnakekeli lomkhulu wemuntfu lophila nesifo sekuwa (epilepsy). PWE kusho bantfu labaphila nesifo sekuwa.

**SIGABA A: LWATI NGEMPHENDVULI**

**A1. Iminyaka: ________**

**A2. Bulili** ☐ Indvodza	☐ Umfati	☐ Lobunye bulili

**A3. Simo semshado** ☐ Akakashadi	☐ Ushadile	☐ Umfelokati/Umfelwa	☐ Udivosile

**A4. Lizinga lemfundvo** ☐ Kute imfundvo lehlelekile	☐ Imfundvo lesisekelo	☐ Imfundvo lesemkhatsini	☐ Imfundvo lephakeme

**A5. Simo sekusebenta** ☐ Uyasebenta	☐ Akasebenti	☐ Uyatisebentela	☐ Ungumfundzi

**A6. Budlelwane nemuntfu lophila nesifo sekuwa** ☐ Umtali	☐ Umnakabo/Dzadzewabo	☐ Umlingani	☐ Lokunye: __________

**A7. Sikhatsi sekunakekela** ☐ Ngaphansi kwetinyanga leti-6	☐ Tinyanga leti-6–umnyaka munye	☐ Umnyaka lo-1–3	☐ Ngetulu kweminyaka le-3

**MPIMO WA TINHLAMULO / SEKALA SA DIKARABO / SIKALI SETIMPHENDVULO**

Kulemisho lelandzelako, khombisa lizinga lovumelana ngalo nayo. Khetsa imphendvulo yinye kumusho ngamunye.

1 = Angivumi nakancane

2 = Angivumi

3 = Angitsatsi luhlangotsi

4 = Ngiyavuma

5 = Ngivuma kakhulu

**SIGABA B: IMIBONO NEKUHLUKUNYEZWA LOKUHLOBENE NESIFO SEKUWA**

| **Inombolo** | **Umusho** | **1** | **2** | **3** | **4** | **5** |
| --- | --- | --- | --- | --- | --- | --- |
| S1 | Ngitiva nginemahloni nangabe bantfu bati kutsi lilunga lemndeni wami linesifo sekuwa. | ☐ | ☐ | ☐ | ☐ | ☐ |
| S2 | Bantfu labaphila nesifo sekuwa baphatfwa ngendlela lengakalungi emphakatsini wakitsi. | ☐ | ☐ | ☐ | ☐ | ☐ |
| S3 | Ngiyakugwema kukhuluma nalabanye ngesifo sekuwa kute ngingahlulelwa. | ☐ | ☐ | ☐ | ☐ | ☐ |
| S4 | Bantfu emphakatsini wakitsi bakholelwa kutsi sifo sekuwa sibangwa buloyi. | ☐ | ☐ | ☐ | ☐ | ☐ |
| S5 | Ngikholelwa kutsi sifo sekuwa sisifo sekulashwa njengaletinye tifo. | ☐ | ☐ | ☐ | ☐ | ☐ |

**SIGABA C: UMTFWALO WEKUNAKEKELA**

| **Inombolo** | **Umusho** | **1** | **2** | **3** | **4** | **5** |
| --- | --- | --- | --- | --- | --- | --- |
| B1 | Kunakekela lomunye kutsintsa likhono lami lekusebenta noma lekutfola imali. | ☐ | ☐ | ☐ | ☐ | ☐ |
| B2 | Ngitiva ngikhatsele emoyeni ngenca yemtfwalo wekunakekela. | ☐ | ☐ | ☐ | ☐ | ☐ |
| B3 | Kunakekela umuntfu lophila nesifo sekuwa sekutsintse imphilo yami yekuhlalisana nalabanye. | ☐ | ☐ | ☐ | ☐ | ☐ |
| B4 | Ngivame kukhatsateka ngekuphepha kwemuntfu lophila nesifo sekuwa. | ☐ | ☐ | ☐ | ☐ | ☐ |
| B5 | Nginenkinga yetimali ngenca yemisebenti yekunakekela. | ☐ | ☐ | ☐ | ☐ | ☐ |

**SIGABA D: TINGCINAMBA**

| **Inombolo** | **Umusho** | **1** | **2** | **3** | **4** | **5** |
| --- | --- | --- | --- | --- | --- | --- |
| D1 | Bantfu emphakatsini wakitsi bakholelwa kutsi sifo sekuwa siyatsatselana. | ☐ | ☐ | ☐ | ☐ | ☐ |
| D2 | Kumatima kufinyelela tinsita tetemphilo tesifo sekuwa. | ☐ | ☐ | ☐ | ☐ | ☐ |
| D3 | Kuneswela lwati ngesifo sekuwa etikolweni nasetindzaweni tekusebenta. | ☐ | ☐ | ☐ | ☐ | ☐ |
| D4 | Tinkholelo temasiko tiphazamisa kwemukelwa kwebantfu labaphila nesifo sekuwa. | ☐ | ☐ | ☐ | ☐ | ☐ |
| D5 | Emalunga emphakatsi ayesaba kusebentisana nebantfu labaphila nesifo sekuwa. | ☐ | ☐ | ☐ | ☐ | ☐ |

**SIGABA E: KWESEKELWA NESIMO LESISITAKO**

| **Inombolo** | **Umusho** | **1** | **2** | **3** | **4** | **5** |
| --- | --- | --- | --- | --- | --- | --- |
| E1 | Imfundvo yetemphilo iyitfutfukisile indlela sifo sekuwa lesibukwa ngayo emphakatsini wakitsi. | ☐ | ☐ | ☐ | ☐ | ☐ |
| E2 | Emacembu ekwesekela banakekeli ayatfolakala futsi ayasita. | ☐ | ☐ | ☐ | ☐ | ☐ |
| E3 | Baholi betenkholo noma bemphakatsi bakhutsata kufakwa kwebantfu labaphila nesifo sekuwa etinhlelweni nasemisebentini yemphakatsi. | ☐ | ☐ | ☐ | ☐ | ☐ |
| E4 | Ngiyakwati kutfola lwati mayelana nekulawulwa kwesifo sekuwa. | ☐ | ☐ | ☐ | ☐ | ☐ |
| E5 | Kuhlanganyela kwemphakatsi kuyasita ekunciphiseni kuhlambalata bantfu labaphila nesifo sekuwa. | ☐ | ☐ | ☐ | ☐ | ☐ |
