# Supplementary material for: Perceived epilepsy-related support and enabling environments and associated caregiver factors in rural South Africa: a cross-sectional study in Limpopo and Mpumalanga
Source: Front Public Health. 2026 Sep 7;14:1881761. doi: 10.3389/fpubh.2026.1881761 (PMC13590295; doi:10.3389/fpubh.2026.1881761)
Supplement: Supplementary file 3 [file Table_1.docx]

**Supplementary Table S1**

*Item wording, coding and allocation to the final subscales*

Agreement items were coded 1 = strongly disagree, 2 = disagree, 3 = neutral, 4 = agree and 5 = strongly agree. Higher summed scores indicate more of the named construct. No item was reverse-scored in the final revised subscales.

| **Item** | **Dataset variable** | **Exact English wording** | **Code** | **Final allocation** | **Final scoring** |
| --- | --- | --- | --- | --- | --- |
| S1 | STIGMA_S | I feel embarrassed when people know my family member has epilepsy. | 1–5 | Standalone item | Reported individually; excluded from the final summed subscales |
| S2 | STATEMEN | People with epilepsy are treated unfairly in my community. | 1–5 | Standalone item | Reported individually; excluded from the final summed subscales |
| S3 | V11_A | I avoid discussing epilepsy with others to prevent judgment. | 1–5 | Standalone item | Reported individually; excluded from final summed subscales |
| S4 | V12_A | My community believes epilepsy is a form of witchcraft. | 1–5 | Standalone item | Reported individually; excluded from the final summed subscales |
| S5 | V13_A | I believe epilepsy is a medical condition like any other. | 1–5 | Standalone item | Reported individually; excluded from the final summed subscales |
| B1 | BURDEN_O | Providing care affects my ability to work or earn income. | 1–5 | Practical, social and financial burden | Summed without reversal |
| B2 | V15_A | I feel emotionally drained by the responsibility of caregiving. | 1–5 | Emotional and safety-related burden | Summed without reversal |
| B3 | V16_A | Caring for someone with epilepsy has affected my social life. | 1–5 | Practical, social and financial burden | Summed without reversal |
| B4 | V17_A | I often worry about the safety of the person with epilepsy. | 1–5 | Emotional and safety-related burden | Summed without reversal |
| B5 | V18_A | I have financial strain due to caregiving responsibilities. | 1–5 | Practical, social and financial burden | Summed without reversal |
| D1 | BARRIERS | People in my community believe epilepsy is contagious. | 1–5 | Standalone item | Reported individually; excluded from the final summed subscales |
| D2 | V20_A | Health services for epilepsy are difficult to access. | 1–5 | Health-system and community barriers | Summed without reversal |
| D3 | V21_A | There is a lack of epilepsy awareness in schools and workplaces. | 1–5 | Health-system and community barriers | Summed without reversal |
| D4 | V22_A | Cultural beliefs interfere with the acceptance of epilepsy. | 1–5 | Health-system and community barriers | Summed without reversal |
| D5 | V23_A | Community members fear interacting with people who have epilepsy. | 1–5 | Health-system and community barriers | Summed without reversal |
| E1 | FACILITA | Health education has improved how epilepsy is viewed in my community. | 1–5 | Standalone item | Reported individually; excluded from the final summed subscales |
| E2 | V25_A | Support groups for caregivers are available and helpful. | 1–5 | Support and enabling environment | Summed without reversal |
| E3 | V26_A | Religious or community leaders promote the inclusion of PWE. | 1–5 | Support and enabling environment | Summed without reversal |
| E4 | V27_A | I have access to information about epilepsy management. | 1–5 | Support and enabling environment | Summed without reversal |
| E5 | V28_A | Community involvement helps reduce stigma against PWE. | 1–5 | Support and enabling environment | Summed without reversal |

**Final subscale construction**

| **Subscale** | **Items** | **No. of items** | **Possible range** | **Interpretation of a higher score** |
| --- | --- | --- | --- | --- |
| Practical, social and financial burden | B1, B3, B5 | 3 | 3–15 | Greater practical, social and financial burden |
| Emotional and safety-related burden | B2, B4 | 2 | 2–10 | Greater emotional and safety-related burden |
| Health-system and community barriers | D2, D3, D4, D5 | 4 | 4–20 | Greater health-system and community barriers |
| Support and enabling environment | E2, E3, E4, E5 | 4 | 4–20 | Greater support and enabling environment |

Note. The original questionnaire grouped items into stigma, burden, barriers and facilitators sections. The final allocations shown here follow the exploratory psychometric revision used in the manuscript. Items S1–S5, D1 and E1 remain useful descriptive indicators but were not combined because they did not demonstrate adequate internal coherence with a final subscale.
