# Supplementary material for: Perceived epilepsy-related support and enabling environments and associated caregiver factors in rural South Africa: a cross-sectional study in Limpopo and Mpumalanga
Source: Front Public Health. 2026 Sep 7;14:1881761. doi: 10.3389/fpubh.2026.1881761 (PMC13590295; doi:10.3389/fpubh.2026.1881761)
Supplement: Supplementary file 4 [file Table_2.docx]

**Supplementary Table S2**

*Complete item-level exploratory psychometric findings (N = 384)*

**S2.1 Analytical approach**

All 20 agreement items had complete data. Overall sampling adequacy was examined before the final subscales were constructed. Because responses were ordinal, ordinal alpha and McDonald’s omega based on polychoric correlations were considered alongside conventional Cronbach’s alpha. Corrected item–total correlations are Spearman correlations between an item and the sum of the remaining items in its final subscale. One-factor loadings are from principal-axis factoring of the relevant polychoric correlation matrix. The analyses are exploratory and do not constitute definitive instrument validation.

**S2.2 Overall dimensional assessment of the 20 agreement items**

| **Statistic** | **Result** |
| --- | --- |
| Kaiser–Meyer–Olkin statistic | 0.830 |
| Bartlett’s test of sphericity | χ²(190) = 3296.61; p < 0.001 |
| First six eigenvalues | 6.08, 2.04, 1.82, 1.33, 1.11 and 1.02 |
| Complete cases | 384/384 (100%) |

**S2.3 Reliability and one-factor results for the final subscales**

| **Final subscale** | **Items** | **Cronbach α** | **Ordinal α** | **Omega** | **KMO** | **Bartlett test** | **One-factor variance** |
| --- | --- | --- | --- | --- | --- | --- | --- |
| Practical, social and financial burden | 3 | 0.518 | 0.640 | 0.645 | 0.644 | χ²(3) = 146.98; p = 1.18 × 10⁻³¹ | 38.0% |
| Emotional and safety-related burden | 2 | 0.808 | 0.867 | 0.867 | 0.500 | χ²(1) = 336.51; p = 3.68 × 10⁻⁷⁵ | 76.6% |
| Health-system and community barriers | 4 | 0.618 | 0.734 | 0.745 | 0.666 | χ²(6) = 388.56; p = 8.03 × 10⁻⁸¹ | 43.2% |
| Support and enabling environment | 4 | 0.569 | 0.723 | 0.729 | 0.690 | χ²(6) = 321.77; p = 1.76 × 10⁻⁶⁶ | 40.8% |

**S2.4 Item-level results for the final subscales**

| **Final subscale** | **Item** | **Item wording** | **Mean** | **SD** | **Corrected item–total ρ** | **One-factor loading** | **Cronbach's α if deleted** |
| --- | --- | --- | --- | --- | --- | --- | --- |
| Practical, social and financial burden | B1 | Providing care affects my ability to work or earn income. | 2.80 | 1.71 | 0.365 | 0.701 | 0.355 |
| Practical, social and financial burden | B3 | Caring for someone with epilepsy has affected my social life. | 2.69 | 1.63 | 0.327 | 0.534 | 0.455 |
| Practical, social and financial burden | B5 | I have financial strain due to caregiving responsibilities. | 2.84 | 1.76 | 0.330 | 0.602 | 0.438 |
| Emotional and safety-related burden | B2 | I feel emotionally drained by the responsibility of caregiving. | 4.00 | 1.36 | 0.745 | 0.875 | — |
| Emotional and safety-related burden | B4 | I often worry about the safety of the person with epilepsy. | 4.20 | 1.23 | 0.745 | 0.875 | — |
| Health-system and community barriers | D2 | Health services for epilepsy are difficult to access. | 2.06 | 1.32 | 0.306 | 0.457 | 0.642 |
| Health-system and community barriers | D3 | There is a lack of epilepsy awareness in schools and workplaces. | 2.29 | 1.48 | 0.401 | 0.603 | 0.559 |
| Health-system and community barriers | D4 | Cultural beliefs interfere with the acceptance of epilepsy. | 2.09 | 1.34 | 0.505 | 0.785 | 0.468 |
| Health-system and community barriers | D5 | Community members fear interacting with people who have epilepsy. | 2.25 | 1.56 | 0.440 | 0.733 | 0.496 |
| Support and enabling environment | E2 | Support groups for caregivers are available and helpful. | 1.65 | 1.01 | 0.487 | 0.697 | 0.470 |
| Support and enabling environment | E3 | Religious or community leaders promote the inclusion of PWE. | 1.57 | 0.97 | 0.430 | 0.590 | 0.514 |
| Support and enabling environment | E4 | I have access to information about epilepsy management. | 1.60 | 0.99 | 0.497 | 0.754 | 0.424 |
| Support and enabling environment | E5 | Community involvement helps reduce stigma against PWE. | 1.70 | 1.21 | 0.357 | 0.481 | 0.579 |

For the two-item emotional and safety-related burden subscale, the alpha when an item is deleted is undefined because only one item would remain. The corrected item–total coefficient is therefore the inter-item Spearman correlation.

**S2.5 Descriptive findings for standalone items**

| **Item** | **Item wording** | **Mean** | **SD** | **1 (%)** | **2 (%)** | **3 (%)** | **4 (%)** | **5 (%)** |
| --- | --- | --- | --- | --- | --- | --- | --- | --- |
| S1 | I feel embarrassed when people know my family member has epilepsy. | 2.26 | 1.63 | 54.7 | 12.8 | 5.7 | 5.7 | 21.1 |
| S2 | People with epilepsy are treated unfairly in my community. | 2.23 | 1.45 | 46.6 | 19.0 | 13.0 | 7.6 | 13.8 |
| S3 | I avoid discussing epilepsy with others to prevent judgment. | 2.99 | 1.66 | 30.5 | 14.6 | 12.5 | 10.7 | 31.8 |
| S4 | My community believes epilepsy is a form of witchcraft. | 3.23 | 1.47 | 17.2 | 17.7 | 20.3 | 14.6 | 30.2 |
| S5 | I believe epilepsy is a medical condition like any other. | 4.57 | 0.99 | 4.7 | 2.1 | 2.3 | 13.0 | 77.9 |
| D1 | People in my community believe epilepsy is contagious. | 3.45 | 1.68 | 22.9 | 12.5 | 8.9 | 8.1 | 47.7 |
| E1 | Health education has improved how epilepsy is viewed in my community. | 3.67 | 1.66 | 18.8 | 13.5 | 5.7 | 6.0 | 56.0 |

Standalone items were retained for descriptive reporting because they did not form a single internally coherent stigma or acceptability scale. Item S5 was analysed in its observed direction (higher scores indicate stronger biomedical recognition of epilepsy).

**S2.6 Spearman item-correlation matrices within the final subscales**

**Practical, social and financial burden**

| **Item** | **B1** | **B3** | **B5** |
| --- | --- | --- | --- |
| B1 | 1.000 | 0.278 | 0.320 |
| B3 | 0.278 | 1.000 | 0.250 |
| B5 | 0.320 | 0.250 | 1.000 |

**Emotional and safety-related burden**

| **Item** | **B2** | **B4** |
| --- | --- | --- |
| B2 | 1.000 | 0.745 |
| B4 | 0.745 | 1.000 |

**Health-system and community barriers**

| **Item** | **D2** | **D3** | **D4** | **D5** |
| --- | --- | --- | --- | --- |
| D2 | 1.000 | 0.112 | 0.412 | 0.266 |
| D3 | 0.112 | 1.000 | 0.364 | 0.458 |
| D4 | 0.412 | 0.364 | 1.000 | 0.413 |
| D5 | 0.266 | 0.458 | 0.413 | 1.000 |

**Support and enabling environment**

| **Item** | **E2** | **E3** | **E4** | **E5** |
| --- | --- | --- | --- | --- |
| E2 | 1.000 | 0.347 | 0.466 | 0.207 |
| E3 | 0.347 | 1.000 | 0.304 | 0.275 |
| E4 | 0.466 | 0.304 | 1.000 | 0.288 |
| E5 | 0.207 | 0.275 | 0.288 | 1.000 |

**S2.7 Interpretation**

The emotional and safety-related burden subscale demonstrated strong reliability. The ordinal reliability estimates for the health-system, community-barrier, support and enabling-environment subscales were acceptable for exploratory use. The practical, social and financial burden subscale showed modest reliability and should be interpreted cautiously. The short length of the subscales, the instrument's self-developed nature, and the exploratory restructuring limit claims of validation. Further cognitive testing, confirmatory factor analysis, test–retest reliability, and measurement invariance assessment are required.

**Supplementary Table S2.8 Item-level psychometric performance of the study subscales**

| **Subscale and item** | **One-factor loading** | **Corrected item-total correlation** |
| --- | --- | --- |
| **Practical, social and financial burden** |  |  |
| Providing care affects my ability to work or earn an income | .701 | .365 |
| Caring for someone with epilepsy has affected my social life | .534 | .327 |
| I experience financial strain because of caregiving responsibilities | .602 | .330 |
| **Emotional and safety-related burden** |  |  |
| I feel emotionally drained by the responsibility of caregiving | .875 | .745 |
| I often worry about the safety of the person with epilepsy | .875 | .745 |
| **Health-system and community barriers** |  |  |
| Health services for epilepsy are difficult to access | .457 | .306 |
| There is a lack of epilepsy awareness in schools and workplaces | .603 | .401 |
| Cultural beliefs interfere with the acceptance of epilepsy | .785 | .505 |
| Community members fear interacting with PWE | .733 | .440 |
| **Support and enabling environment** |  |  |
| Support groups for caregivers are available and helpful | .697 | .487 |
| Religious or community leaders promote the inclusion of PWE | .590 | .430 |
| I have access to information about epilepsy management | .754 | .497 |
| Community involvement helps reduce stigma against PWE | .481 | .357 |

*Note. Factor loadings were obtained from one-factor principal-axis models based on polychoric correlation matrices. Corrected item-total correlations are Spearman correlations between each item and the sum of the remaining items in its subscale. PWE = people with epilepsy.*

Practical, social and financial burden: KMO = .644; Bartlett’s χ²(3) = 146.98, p < .001.

Emotional and safety-related burden: KMO = .500; Bartlett’s χ²(1) = 336.51, p < .001.

Health-system and community barriers: KMO = .666; Bartlett’s χ²(6) = 388.56, p < .001.

Support and enabling environment: KMO = .690; Bartlett’s χ²(6) = 321.77, p < .001.
